# Implication of Mitochondrial Cytoprotection in Human Islet Isolation and Transplantation

**DOI:** 10.1155/2012/395974

**Published:** 2012-05-07

**Authors:** Yong Wang, Joshua E. Mendoza-Elias, Meirigeng Qi, Tricia A. Harvat, Sang Joon Ahn, Dongyoung Lee, Diana Gutierrez, Hyojin Jeon, Daniel Paushter, José Oberholzer

**Affiliations:** Department of Transplant/Surgery, University of Illinois at Chicago, Chicago, IL 60612, USA

## Abstract

Islet transplantation is a promising therapy for type 1 diabetes mellitus; however, success rates in achieving both short- and long-term insulin independence are not consistent, due in part to inconsistent islet quality and quantity caused by the complex nature and multistep process of islet isolation and transplantation. Since the introduction of the Edmonton Protocol in 2000, more attention has been placed on preserving mitochondrial function as increasing evidences suggest that impaired mitochondrial integrity can adversely affect clinical outcomes. Some recent studies have demonstrated that it is possible to achieve islet cytoprotection by maintaining mitochondrial function and subsequently to improve islet transplantation outcomes. However, the benefits of mitoprotection in many cases are controversial and the underlying mechanisms are unclear. This article summarizes the recent progress associated with mitochondrial cytoprotection in each step of the islet isolation and transplantation process, as well as islet potency and viability assays based on the measurement of mitochondrial integrity. In addition, we briefly discuss immunosuppression side effects on islet graft function and how transplant site selection affects islet engraftment and clinical outcomes.

## 1. Introduction

Since the introduction of the Edmonton Protocol in 2000 [1], human islet transplantation has emerged as a promising therapy for type 1 diabetes mellitus (TIDM) and is the only minimally invasive therapy able to achieve glycemic control without exogenous insulin. As a cell therapy, islet transplantation is a multistep process involving pancreas organ procurement and preservation, tissue digestion and dissociation, islet purification, cell culture, islet transplantation via the hepatic portal vein of the recipient, and maintenance of the graft by nonsteroidal immunosuppressant regiments. Successful islet transplantation is dictated by the cumulative success of the aforementioned steps. To date, islet transplantation has been shown to have variable success in both short- and long-term insulin independence [2–5] and much of this variability is associated with factors from both the organ donor and recipient. In order to maximize the success rate of achieving insulin independence, considerable efforts have been focused on the following aspects: (i) development of a suitable organ preservation solution; (ii) standardization of good manufacturing principles (GMP) in islet isolation; (iii) development of an accurate potency test that can predict islet graft function prior to transplantation (as outlined by requirements for an FDA Biologics License); (iv) development of a new immunosuppression regimen with less islet graft toxicity; (v) improvement of islet graft survival via *ex vivo* and *in vivo* manipulation; (vi) selection of a more ideal transplant site.

While we often focus on getting more islets from each islet isolation, one of the top priorities that should not be neglected is the attainment of better quality islets with preserved *β*-cell function and viability. Located in the cytoplasm, the main function of the mitochondria is to provide energy for the cell in the form of ATP. Cells use this energy to perform the work necessary for cell survival and function. Moreover, mitochondria are also involved in other cell processes such as cell growth, division, and apoptosis. Pancreatic islets consist of a cluster of 1000–2000 cells and ranging in size between 50–400 *μ*m in diameter. Composing 1-2% of the pancreas mass, each islet consists of at least five different cell types: *α*-cells (15–20% of islet mass) producing glucagon; *β*-cells (65–80% of islet mass) producing insulin and amylin; *δ*-cells (3–10% of islet mass) producing somatostatin; PP cells (3–5% of islet mass) producing pancreatic polypeptide; *ε*-cells (<1% of islet mass) producing grhelin. Specifically for *β*-cells, mitochondria play a key role in glucose-stimulated insulin secretion, not only by providing energy in the form of ATP to support insulin secretion, but also by synthesizing anapleurtoic metabolites that can act, both intra- and extramitochondrially, as factors that couple glucose sensing to insulin granule exocytosis. ATP on its own, and possibly modulated by these anapleurotic coupling factors, triggers closure of the ATP-sensitive potassium channel, resulting in membrane depolarization that increases intracellular calcium to cause insulin secretion [6–9]. 

Recently, the roles of mitochondria in cytoprotective applications involving ischemia-reperfusion (I/R) injury have been acknowledged and continue to be actively investigated using chemical and physical means during both islet isolation and on posttransplanted islet grafts. However, no systematic review has been done in this respect. In this review, we will examine current and past research from the last ten years on the role of mitochondria and mitoprotective strategies that have been applied in each phase of the islet isolation process.

## 2. Mitochondria and Mitoprotection in Pancreas Organ Preservation and Storage

While initial results of human islet transplantation using the Edmonton Protocol are promising, the need for a large quantity of islets to achieve insulin independence from multiple organs per patient creates an obstacle to the application of human islet transplant as a routine clinical procedure. Pancreas preservation and storage is the first step in the islet isolation process and has a critical impact on islet yield and quality. During the isolation process, only a fraction of the islets in a whole pancreas can be successfully isolated with good function and viability; this is especially true for organs with a prolonged cold ischemia time.

Ischemia injury has been shown to significantly decrease the quantity and quality of islets that can be isolated from a pancreas [10–12]. Hypoxia and ischemia, during the process of pancreas procurement and storage, triggers a cascade of cell signaling pathways that compromise islet viability and function [13–15]. University of Wisconsin (UW) and Histidine-Tryptophan-Ketoglutarate (HTK) solutions are the two most commonly used organ preservation solutions intended for islet isolation. Both are designed to protect the organ from ischemia-related cell injury. As a gold standard, UW solution is the first solution thoughtfully designed for use in organ transplantation and the first intracellular-like preservation medium. UW solution contains metabolically inert substances (lactobionate, raffinose, and hydroxyethyl starch) as a glucose substitute that maintains native osmotic pressure, thereby preventing cellular edema. In addition, UW solution also adds free radical scavengers along with steroids and insulin. In contrast, the composition of HTK is similar to that of the extracellular fluid and is developed based on the principle of deactivating and suspending of cellular organ function by withdrawal of extracellular sodium and calcium. Together with intensive buffering of the extracellular space by means of histidine/histidine hydrochloride, the period during which organs will tolerate prolonged interruption of oxygenated blood may be increased. However, both solutions do not prevent the deleterious effects of hypoxia and ischemia *per se*, especially for prolonged cold storage of the pancreas [16–18].

Enormous attempts have been made to reduce ischemia injuries through the oxygenation of preservation solution. Perfluorochemicals (PFCs) are one of the chemicals that have been developed so far with the most significant impact on islet isolation. The PFCs are cyclic or aliphatic hydrocarbon molecules in which all the hydrogen atoms have been replaced with fluorine. PFCs are a good solvent for gases and approximately 40 mL of oxygen can be dissolved in 100 mL of 90–95% PFC solutions. This makes the compound attractive as a vehicle to deliver oxygen and other gases *in vivo* and *in vitro*. PFCs were first induced by Kuroda et al. for whole pancreas preservation in 1988 [19–21] as a two-layer storage method (TLM) in combination with UW solution and later adopted in pancreas preservation for islet isolation. Since then, more than 50 research papers have been published in both human and animal models and the impacts of PFCs on islet isolation outcomes have been reviewed [22–27]. In the following section, we will briefly address the major findings from these studies and discuss the possible reasons for the observed discrepancy in success and failure for islet isolation using PFCs and summarize these observations in Table 1, along with additional oxygen carriers used for islet isolation.

In their early studies, Matsumoto and Kuroda showed that when PFCs were used within the TLM protocol, they demonstrated significant increases in islet yield, function, and viability. In addition, PFCs extended preservation times [28–30] when compared against UW solution alone. The TLM was then immediately and widely adopted early by several renowned islet programs, which demonstrated similar results [10, 31–33] and later adopted by others [34, 35]. One study demonstrated that islets isolated using the TLM had an improved energetic profile with higher ATP content and ATP/ADP ratio, as wells as 40% less peroxidative damage, indicating the mitochondria as a likely target in a potential mechanism [36]. In 2006, Ramachandran et al. analyzed the expression of pro- and antiapoptotic genes in the islets isolated using the TLM, which showed a significant increase in the expression of inhibitor of apoptosis (IAP) and survival; accompanied by decreases in the expression of BAD, BAX, and caspases (caspase-2, -8, and -9). The improved islet yield by PFCs was linked to the inhibition of apoptosis mediated by an undescribed mitochondrial pathway [37]. However, more recently, several large-scale studies and a retrospective meta-analysis indicated that the beneficial effect of the TLM on islet isolation and clinical transplantation outcomes are minimal as PFCs may only improve the preservation of marginal organs [38–42]. Many contribute this finding to the poor capability of PFCs to promote oxygen penetration into the whole pancreas as PFCs cannot be flushed into the pancreatic vascular system either with or without UW solution due to its poor water solubility. In 2009, Matsumoto et al. reanalyzed their previous data and contributed the observed discrepancy to the lack of experience of organ procurement teams [27] since a standardized protocol was not widely accepted across all centers.

To date, there have been few other oxygen carriers that have been tested for their effects on pancreas preservation intended for islet isolation, mainly due to their low oxygen carrier capability, poor water solubility, and poor biocompatibility. In the pig pancreas model, oxygenated perfluorodecalin (PFD) was used at various temperatures showing that pig pancreas oxygenation at 20°C increases islet ATP generation; however, the islets were found to have compromised insulin secretion in response to glucose stimulation [43]. In a human islet study, F6H8S5, a newly developed oxygen carrier composed of F6H8 (perfluorohexyloctane) and silicone oil polydimethylsiloxane 5, was compared to PFD, showing comparable results linking the optimization of ATP production during the cold storage period [44].

In a warm-ischemia rat model, oxygenated poly SFH-P (polymerized and stroma-free hemoglobin-pyridoxalated) was applied to the pancreas prior to enzyme digestion, showing that the islets isolated from a poly SFH-P treated pancreas had improved *β*-cell viability and mitochondrial potential changes in response to glucose stimulation. In addition, O_2_ delivery by the poly SFH-P did not increase glutathione (GSH) levels (an indicator for oxidative stress) or malondialdehyde (MDA) levels (an indicator for oxidative injury) [45]. These results supported the notion that there was a mitoprotective effect associated with the Poly SFH-P treatment. However, it is worth noting that other groups did not further confirm this study either in rodent or human islet isolation process due to discontinuation of the product.

There have been multiple attempts to supplement UW solution with other mitoprotective agents such as trophic factors [46, 47], mitochondria-targeted antioxidant mitoquinone [48], carvedilol (a non-selective *β* blocker/*α*-1 blocker) [49], epidermal growth factor (EGF), and insulin-like growth factor-1 (IGF-1) [50, 51]. However, the benefits of these chemicals have not been verified by systematic studies or used in pancreas organs intended for islet isolation.

Furthermore, a specific problem linked to pancreas organ preservation is the leakage of zymogens from the exocrine pancreas. Within the pancreas, serine proteases exist in the form of an inactive proenzymes and are secreted from the pancreas as trypsinogen, chymotrypsinogen, and proelastase. The activation pancreatic proteases during isolation has been observed and found to be caused in part by crude impurities in collagenase preparations [52]. The resulting cell death during the isolation process releases immunogenic intracellular proteases that prompt the generation of free radicals in the mitochondria and trigger activation of macrophages to produce proinflammatory cytokines. In addition, Heiser et al. reported that there was a strong increase in trypsin activity levels throughout the digestion phase and that these increased levels correlated with poor islet yields and function [53]. It has also been shown in several studies that the addition of protease inhibitors, such as Pefabloc and *α*_1_-antitrypsin (A1AT) in UW and HTK solutions, as well as used as in the TLM, can increase islet yield and function by effectively inhibiting this uncontrolled protease activity [32, 54–56]. However, a recent large-scale study showed that Pefabloc actually did not improve the overall islet yields. In addition, in one study, it was found in that pancreas preserved in the TLM may provide even better transplant quality islets in the absence of Pefabloc [57].

In summary, although some promising studies have shown the beneficial mitoprotective effects for islets by oxygenation and chemical addition to organ preservation solutions, the involved underlying mechanisms and their overall impacts remain controversial and grounds for further study. In addition, since the introduction of the Edmonton Protocol, islet yield from one pancreas remains low with an average of 300,000–400,000 IEq depending on center expertise. Organ preservation solutions such as UW or HTK are still considered the most effective solution for preventing ischemic injury until other alternatives are available that specifically address the needs unique to pancreata intended for islet transplant.

## 3. Mitochondria and Mitoprotection in Islet Isolation and Culture

Since reperfusion injury is one of the primary factors associated with islet cell death during islet isolation, there have been extensive investigations dedicated to minimizing the effects of reperfusion injury in order to improve islet isolation outcomes. Reperfusion-related cell damage is due in part to the inflammatory response of islet tissues mediated by the release of inflammatory factors including interleukins and reactive oxygen species (ROS). Although the mechanism is not fully characterized, both inflammatory factors and ROS intertwine as either initiator or effector in an inflammatory cascade during islet isolation that may cause *β*-cell dysfunction and death. During reperfusion and reoxygenation, significantly increased levels of ROS can degrade cell and capillary membranes that cause additional release of free radicals and act indirectly to initiate redox-signaling pathways leading to apoptosis. In addition, reoxygenation can restore ATP levels that may cause an increase in calcium uptake by the mitochondria, resulting in massive calcium overload and destruction of the mitochondria.

Oxidative stress has been shown to play a pivotal role in cell injury during the islet isolation and transplantation processes [106–108]; therefore, blocking oxidative stress should have a beneficial impact on transplantation outcomes. To date, several antioxidants have been used to protect islet cells from oxidative injury during the isolation and culture period. Herein, we will briefly discuss these findings.

Bottino et al. demonstrated that activations of nuclear factor-kappaB (NF-*κ*B) and poly (ADP-ribose) polymerase (PARP), two of the major pathways responsible for cellular responses to stress, occur in islet cells during the isolation procedure and precedes cellular dysfunction and demise including the disruption of mitochondrial membrane potentials (MMPs); mitochondrial permeability transition pore (MPT) formation; and an intracellular increase of accumulating ROS. NF-*κ*B-dependent reactions, such as the production and release of interleukins (IL-6 and IL-8) and macrophage chemoattractant protein-1 (MCP-1), were observed days after the isolation procedure. Proinflammatory responses were even more pronounced when islets were cultured under specific conditions which mimicked isolation stress and correlated well with higher islet cell loss and impaired secretory function [58]. In addition, Bottino et al. showed that early interventions aimed at reducing *β*-cell oxidative stress through the use of the catalytic antioxidant probe AEOL10150 (manganese [III] 5,10,15,20-tetrakis [1,3,-diethyl-2imidazoyl] manganese-porphyrin pentachloride [TDE-2,5-IP]) can effectively reduce DNA binding of NF-*κ*B and the subsequent release of cytokines, chemokines, and PARP activation in islet cells; thus resulting in higher survival and better insulin release when compared to controls [58]. A similar study conducted by the same authors demonstrated that the application of two SOD mimetics (AEOL10113 and AEOL10150) can also protect human islets from oxidative stress, showing a significantly higher viable islet mass and better *in vivo* islet graft function using a marginal islet mass transplant model [59].

GSH (glutamate-glycine-cysteine) is one of the most important nonenzymatic antioxidants available; however, it is not cell permeable. Cells can synthesize GSH from its precursors, such as glutamine. The importance of glutamine for cell survival and proliferation *in vitro* was first described by Eagle et al. in 1956 [60]. The cytoprotective and antiapoptotic effects of glutamine have been demonstrated in intestinal epithelial cells by Evans et al. [61] and also in pancreatic islets showing that GSH increases *β*-cell insulin secretion [62–64]. In both human and rodent models, intraductal delivery of glutamine into the pancreas prior to digestion can increase GSH levels, reduce MDA levels, and reduce the number of apoptotic cells. In addition to an improved islet yield, the percentage of nude mice rendered normoglycemic with glutamine-treated islets was higher than the controls and time to reach normoglycemia was decreased from average of  7.3 ± 3  days to 1.83 ± 0.4 days [65, 66].

Although most antioxidant compounds have a wide extracellular and intracellular distribution, they often fail to accumulate within the mitochondria and require conjugation with lipophilic cations for mitochondrial targeting. Their ability to enter the mitochondria and accumulate within the matrix also depends on the inner mitochondrial membrane potential (MMP) and its proton gradient; a feature which may change depending on the metabolic status of cells. This requirement may limit their capacity to permeate depolarized cells. Moreover, the accumulation of these antioxidant cations within the mitochondrial matrix can lead to dissipation of inner MMP, an event that often leads to cell death. As a result, these agents exhibit narrow therapeutic dose ranges. To combat this, genetic strategies are being explored to bolster the antioxidant defense of islets. *Ex vivo* transfer of the manganese superoxide dismutase (MnSOD) gene to mouse islets has extended islet graft function in autoimmune diabetic mice. It has been observed that the islets from mice that overexpress GSH peroxidase and the two isoforms of SOD improve blood glucose control in a marginal islet mass model.

A point of concern to its clinical application is that it has been demonstrated that viral delivery systems can pose potential oncogenic risks, compromise islet function, and increase immunogenicity; the latter being especially concerning given the pathophysiological setting of T1DM. Recently, two research groups have demonstrated that polyvalent gold nanoparticles densely functionalized with covalently immobilized DNA oligonucleotides (AuNP-DNA) have a high penetrative capacity for islet cells which can reach the islet cores of both mouse and human islets with no evidence of toxicity; demonstrating that islet function is preserved well both *in vitro *and* in vivo* as compared to control. One study showed that AuNP-treated islets have normal mitochondrial response kinetics when stimulated by glucose, in conjunction with preserved calcium influx and insulin secretion when compared to control [109]. The other study indicated that AuNP-DNA conjugated with antisense eGFP reduced eGFP expression in MIP-eGFP islets (mouse insulin promoter controlled eGFP) [110]. AuNP and/or AuNP-conjugates may represent a new generation of nucleic acid-based therapeutic platforms aimed at improving islet engraftment, survival, and long-term *in vivo* function. It is expected that more investigations into the use of nanoparticles for cargo delivery to islets will follow to confirm this observation while improving this functional delivery system.

In addition to using physical, chemical, and viral platforms for delivery of antioxidative cargo, molecules that have good penetrative ability have also been investigated. SS-31 (D-Arg-2′,6′-dimethyltyrosine-Lys-Phe-NH_2_), a novel water-soluble antioxidant peptide, is one of a few molecules tested that have demonstrated the ability to penetrate into the core of islets and localize in the mitochondria [67]. In addition, SS-31 preserves MMPs, reduces islet cell apoptosis, increases islet cell yield, and improves posttransplantation function as demonstrated by a prompt and sustained normoglycemia; whereas untreated islet graft recipients remained diabetic. One study has demonstrated that SS-31 can inhibit, with minimal cellular toxicity, mitochondrial swelling, oxidative cell death, and I/R injury of cardiac and neuronal cells [68]. However, to date there are no published reports of the effect of SS-31 in transplanted human islets.

The effector mechanisms by which cytokines induce *β*-cell death may also encompass both nitric oxide (NO) dependent and independent pathways [69]. In rodent islets, interleukin-1 beta (IL-1*β*) alone can induce iNOS expression and the resultant NO production impairs *β*-cell function by oxidation of mitochondrial aconitase (m-con) which leads to diminished glucose oxidation and ATP production [70]. Steer et al. demonstrate that this NO-mediated pathway of *β*-cell death is primarily necrosis mediated by the release of the high-mobility group box 1 protein (HMGB1) [71]. In contrast, human islets appear to be more resistant to cytokine induced NO production and the primary mechanism of dysfunction may be the result of mitochondrial ROS production and activation of proapoptotic caspase enzymes [72]. A variety of specific and nonspecific inhibitors of reactive nitrogen and oxygen species have been evaluated for their effects on islets in culture and *in vivo* posttransplant. Inhibition of iNOS by NG-monomethyl-L-arginine (NMMA) or aminoguanidine (Pimagidine) in culture can block NO production in both rodent and human islets [73]. Treatment with N-acetyl cysteine or glutathione peroxidase can enhance the antioxidant capacity of islet *β*-cells [74, 75].

When human pancreatic islets are treated with a cytokine combination of IL-1*β*, tumor necrosis factor-alpha (TNF-*α*), and interferon-gamma (IGN-*γ*) for 72 h, a significant increase in the amount of cell death was observed by TUNEL (terminal deoxynucleotidyl transferase dUTP end labeling) and cell death detection [68]. Furthermore, this study demonstrated that this cell death was associated with apoptosis and mitochondrial swelling. A possible link has been suggested between peripheral benzodiazepine receptors (PBR), also known as translocator protein (TSPO), which implicates a specific mitochondrial permeability transition pore and tissue damage associated with the production of inflammatory mediators.

Various vitamins (D3, E, Riboflavin, and C) have also been used as antioxidants *in vitro* and *in vivo* and show protective benefits including increased insulin secretion, higher islet cell viability, increased insulin gene expression, and reduced lipid peroxidation. Since these results have been reviewed previously [76], in this review we will not further discuss the findings.

Recently, it has been hypothesized that a better strategy could be to block the initiation of the inflammatory pathways triggered by the interaction of IL-1*β* with its cellular receptor (interleukin-1 receptor, IL-1R). The effectiveness of the IL-1R antagonist (Kineret, (Anakinra)) in blocking the proapoptotic and necrotic effects of exogenous IL-1*β*, TNF-*α*, and IFN-*γ* on cultured rat islets has been investigated. Anakinra is a U.S. FDA approved drug used as an anti-inflammatory therapy in clinical trials of human subjects with rheumatoid arthritis [77] and type 2 diabetes mellitus (T2DM) [78]. It is a human recombinant, nonglycosylated form of the human interleukin-1 receptor antagonist (IL-1ra) consisting of 153 amino acids with a molecular weight of 17.3 kDa. The biologic activity of Anakinra derives from competitive inhibition of IL-1*β* binding to the interleukin-1 type I receptor (IL-1RI). In several animal models and *in vitro* human islet studies, blocking IL-1*β* receptor (IL-1R) has shown an effective inhibition of activation of IL-1*β*-dependent inflammatory pathways [79], enhancement of islet engraftment [80] and islet graft function, and attenuation of amyloid polypeptide-induced proinflammatory cytokine release [81]. All of these results support its feasible application to human islets *in vivo* as a posttransplant therapeutic regiment.

Two studies conducted by one group have demonstrated the application of either pan-caspase inhibitor (N-benzyloxycabonalyl-Val-Ala-Asp-fluoromethyl ketone [Z-VAD-FMK]) or more selective caspase inhibitor (EP1013 [zVD-FMK]) during postisolation culture and *in vivo* treatment can reduce islet loss following prolonged culture and reverse diabetes following implantation of a marginal human islet mass (80–90% reduction) into mice [82, 83]. Several similar studies have also demonstrated the benefits of caspase inhibitors for islet transplant [84, 111].

Some studies have demonstrated a significant beneficial effect of recombinant human prolactin (rhPRL) on *β*-cell proliferation, islet secretion, cytoprotection, islet engraftment, and function of the transplanted islets by revascularization and *β*-cell survival [85–87]; however the underlying mechanism is unclear. One recent study has shown that cytoprotection may involve an increase in BCL2/BAX ratio and inhibition of several caspases such as 8, 9, and 3 [88].

The signal transduction pathway of c-Jun NH_2_-terminal kinase (JNK), a member of the stress-activated group of mitogen-activated protein kinases (MAPKs), is preferentially activated in response to islet isolation process, oxidative stress, and cytokine toxicity [89]. It has been reported that an IL-1*β* storm caused by brain death significantly activates JNK and reduces islet yield, viability, and function both *in vitro* and *in vivo* after transplantation [90]. It has also been shown that islet injection into the hepatic portal vein system leads to a strong activation of JNK [91]. The application of JNK inhibitor during islet culture and transplantation can prevent islet loss and *in vivo* graft function [91, 92]. In addition, a JNK inhibitor has also been also added to pancreas preservation solution showing a strong inhibition of JNK which prevented apoptosis immediately after isolation [93].

Previously, we discussed that the activation of trypsin and other endogenous pancreatic zymogens contribute to decreased islet yields via the uncontrolled proteolysis of collagenase and the prolongation of digestion times. Attempts to use Pefabloc during the isolation process instead of organ preservation phase have been made, showing that the Pefabloc can inhibit serine protease activity throughout the tissue digestion process; but, with controversial results on islet yield and function [94–96]. In addition, two research papers recently published by the same group show that when human islets were cultured or perifused with Pefabloc postisolation, insulin secretion of human islets in response to glucose stimulation was compromised, suggesting that the Pefabloc is not suitable for human islet isolation [97, 98].

Summarized in Table 2, are the major antioxidative and anti-inflammatory chemicals used in pancreas preservation, islet isolation, and islet culture.

## 4. Evaluation of Mitochondrial Integrity for Human Islet Function and Viability Assays

The U.S. Food and Drug Administration (FDA) defines the preparation of the islet product as a biological drug; therefore, islet products for transplant need to be prepared under FDA-approved guidance before islet transplant can be approved as a clinical therapy. Despite standardization and application of current good manufacturing practices (cGMPs) in the islet isolation process, lot-to-lot variability still cannot be avoided. To reduce the risk of transplanting low-quality islets, appropriate product release tests are needed. While tests for sterility, identity, and purity are well established, so far no reliable assessment of islet potency is available prior to transplant. This continues to be one of the key variables associated with clinical transplant outcomes.

The existing standard assays used to assess islet function and viability include static glucose-stimulated insulin stimulation (GSIS) for potency, and inclusive and exclusive dyes to stain membrane integrity for viability. Both of these techniques have low predictive values and do not correlate well with clinical transplant outcomes [112–116]. Presently, clinicians determine the suitability of a given islet preparation mainly based on both subjective measurements (e.g., morphology) and islet cell mass (IEq, a volumetric quantification of islet mass). The underlying reasons for the poor predictive value of GSIS are less clear; however, there are several possible explanations. Static GSIS only measures “bulk” insulin release from an islet preparation under extreme conditions (16.7 mM and 60 min glucose exposure), completely ignoring the dynamic physiological nature of *β*-cell insulin secretory kinetics in response to glucose. In addition, the “bulk” insulin data does not provide any useful information of the key stimulus-coupling factors that are necessary for controlling and regulating both insulin secretion and viability *in vivo*. For example, mild stress during pancreas preservation and islet isolation process can lead to temporary insulin degranulation and leakage caused by cell membrane damage, even though the islets are still viable. Therefore, a low GSIS may not indicate irreversible loss of function, as the temporarily impaired islet cells may recover when transplanted into a recipient. Likewise, a high GSIS result may be caused by insulin degranulation via cell membrane damage. 

Islet size is another factor that can cause variable GSIS results. Unlike *in situ*, the isolated islet's sensing of ambient glucose changes is totally dependent on the passive diffusion of glucose [99, 114]. Since smaller islets have a larger surface area, a better GSIS would be expected [100, 117] in a population of smaller islets. In clinical practice, IEq is used as a determining factor in deciding the suitability of a given islet preparation when human islet potency is less defined or hard to quantify. It is a general consensus that, in order to reverse diabetes in type 1 diabetes recipients, at least 5,000 IEq per kilogram of recipient body weight are needed for one transplant. Paradoxically, larger size islets contribute more IEq but release less insulin which is a likely explanation of why islet graft success does not correlate strongly with transplanted islet mass. 

On the contrary, the *in vivo* potency assay which evaluates islets by transplanting human islets into immunodeficient nude mice correlates extremely well with clinical transplant outcomes and is currently the gold standard for evaluating islet potency. However, this *in vivo* analysis takes several weeks to complete and, therefore, only provides a retrospective indication of islet function that renders this assay impractical as a pretransplant assessment in the time critical clinical setting [101, 103, 118, 119].

To address this problem, a variety of *in vitro* tests have been investigated and developed in order to assess islet potency and viability prior to islet release for transplant, including: measuring the oxygen consumption rate (OCR) [104, 114, 115, 120, 121]; the quantification of reactive oxygen species (ROS) [116]; and ADP/ATP ratios [105, 122]. In addition to being retrospectively useful and less predictive, these assays have multiple limitations. To begin with, most OCR and ROS assays are conducted statically as a single parameter. Additionally, OCR and ROS assays are less *β*-cell specific, as an islet has at least five different cell types and *β*-cells only make up 65–80% of a human islet cell population. Without a weighted analysis of ROS for each individual cell group, this information is confounded; and even then the measurements may be artefactual as islets are one physiologic and metabolic functional unit. Recently, more and more evidence has demonstrated that chemical and/or ion communication among *β*-cells or between *β*-cells and *α*-cells is important for the regulation of insulin secretion [123, 124]. In addition, gap junctional complexes between adjacent islet cells are also needed to facilitate intraislet cell-cell communications and coordination for hormonal output [102]. Therefore, others often challenge the accuracy of ADP/ATP assay since the assay requires islet dissociation. A comprehensive review on these assays has been recently published [113]. Herein, we will briefly discuss the assays measuring mitochondrial integrity for islet potency and viability. In Table 3, we summarized the strengths and limitations of these assays.

In the search for a more reliable and *β*-cell specific potency assay, the measurement of mitochondrial integrity has been emerging as an alternative assay in the form of a single parameter or combined with other parameters for islet viability and potency.

Zinc is highly concentrated in *β*-cells but much less concentrated in other islet cell types. Zinc plays an important role in the packaging insulin granules as it is firmly established as an integral coordinating atom of the insulin crystal as a 2-Zn-insulin hexamer. Recently, Newport Green (NP), a less-toxic zinc-sensitive fluorescent probe, has been used for the identification and purification of human *β*-cells [125]. In 2005, Itchii et al. reported a novel combined analytical method in which *β*-cells were first specifically identified using NP labeling and sorted by laser scanning cytometry (LSC). NP^+  ^positive cells were then evaluated for viability using TMRE (tetramethylrhodamine ethyl ester), a mitochondrial membrane potential fluorescence probe [126]. The data presented in the study strongly suggest that analysis of *β*-cell viability by TMRE is of critical value for the prediction of transplant outcomes in the immunodeficient mouse model. Furthermore, the study finds that DNA-binding viability exclusion dyes do not always correlate with *in vivo* graft function; a strong argument suggesting the inadequacy of inclusion/exclusion dyes as a predictive assay of long-term islet viability. These findings are further confirmed by other studies [127]. In addition, TMRE used in conjunction with FluoZin-3, another zinc-specific probe but with a higher affinity (*K*_*D*_ = 15 nM) and higher quantum yield, can also be used to assess *β*-cell viability using flow cytometry. The functionality of *β*-cells is determined by the retention of the MMP indicator in conjunction with TMRE in FluoZin-3^+^ *β*-cells [128, 129]. In other studies, the MMP measured by the mitochondrial JC-1 fluorescence probe are used in parallel to correlate changes in luminol-measured ROS, showing a decrease in the percentage of cells with normal mitochondrial polarity with a low responsive index in both glucose- and rotenone-stimulated islets. Regression analysis shows the rotenone stimulation index is significantly correlated with the percentage of islet cells with polarized mitochondria [116, 130].

While these results are promising, the aforementioned assessments of mitochondrial integrity measure a static value of *β*-cell energetic status with only retrospective values since these assays may take days to conduct and cannot predict islet graft function at the time of transplant. In addition, as an enzymatic dissociation of the islets is involved, the assays' methodology is often questioned for introduction of artefacts, such as selective damage or and loss of to the *β*-cell population. Furthermore, most of these assays require more sophisticated laboratory setups, such as FACS, confocal microscope, and laser scanning cytometry. However, these findings strongly suggest that a testing method that has the capability of measuring dynamic changes in mitochondrial potential kinetics in whole islets in response to insulin secretagogues (such as glucose), in parallel with other assays that measure key insulin stimulus-secretion coupling factors, would be useful in determining islet function prior to transplantation. In order to achieve this type of a test, it is necessary to use microfluidic technology.

Microfluidic technology has been used for a wide range of analytical applications and recently these techniques have been adopted to develop versions of islet perfusion apparatuses, integrated with either single or multiple analysis tools for islet and *β*-cell studies [131–133]. Microfluidic technology has a number of advantages over conventional techniques in that smaller amounts of reagent and bioanalyte are required, it is simple to create and maintain an experimental microenvironment, and microfluidic assessment allows for the easy integration of analytical tools such as optical and electrical assays. In addition, the fabrication techniques are favorable for portability and economic mass production of highly elaborate devices. Recently, a microfluidic-based perfusion setup has been developed for the simultaneous imaging of mitochondrial potentials changes and calcium influx as well as insulin release kinetics of whole islets in response to glucose stimulation [134, 135]. This mitochondria-based islet assay is associated with the following features: (i) multiparametric assay of key insulin stimulus-secretion coupling factors including intracellular calcium signaling, mitochondrial potentials changes, and insulin secretion kinetics; (ii) real-time whole islet assay with no islet fixing or dissociation is needed; (iii) *β*-cell specific since islets are evaluated in response to stimulation by insulin stimulators; (iv) sufficient spatiotemporal resolution of all measured parameters. Although the system shows promising results, a large-scale evaluation of human islet products is needed in conjunction with data correlating results to *in vivo* graft function in both animal and human models. This work is currently under way following a clinical trial and is expected to validate a mathematical model for an islet functional index (IFI) score.

In spite of various assays available, a conclusive potency test still needs to be developed and verified for universal acceptance by the islet transplant community. Ideally, this potency test needs to be real-time, *β*-cell-specific, sensitive, reliable, robust, and have practical applicability.

## 5. Impact of Mitochondria on Selection of Immunosuppression Regiment and Transplant Site

Besides the infusion of a sufficient number of high-quality islets and careful recipient selection of patients with normal kidney function and low insulin requirements, another important factor for the success of the Edmonton Protocol is the development of a new steroid-free immunosuppressive therapy in combination of sirolimus (rapamycin, an mTOR inhibitor) and low dose of tacrolimus (FK506, a calcineurin inhibitor), and induction with daclizumab (an anti-IL2-receptor monoclonal antibody).

It has been suggested in the past that posttransplant islet graft failures can be caused by several reasons including initial failure of islet engraftment, inflammatory response at the transplant site, alloreactive (rejection) or autoimmune response, and immunosuppressive drug-induced *β*-cell toxicity [136]. Although their long-term side effects are still not fully known, immediate side effects of immunosuppressive drugs include mouth sores, gastrointestinal disturbance (stomach upset and diarrhea), high cholesterol levels, hypertension, anemia, fatigue, decreased white blood cell counts, decreased kidney function, and increased susceptibility to bacterial and viral infections. Taking immunosuppressive drugs also increases the risk of tumors and cancer. Several studies have systemically reviewed each of these aspects; this review will only briefly address a few of the findings of the effects of immunosuppressants in relation to islet mitochondrial-based cytoprotection.

Although extensive studies have investigated the effects of sirolimus and tacrolimus on islets *in vitro* and *in vivo*, as well as in the islet transplant patients, conflicting results have been reported. Sirolimus inhibits the mTOR (the mammalian target of rapamycin) pathway by directly binding mTOR Complex 1 (mTORC1). It has been shown that the mTOR signaling pathway integrates both intracellular and extracellular signals and serves as a central regulator of cell metabolism, growth, proliferation, and survival. It has also been demonstrated that mitochondrial metabolism and biogenesis are both regulated by mTORC1. Through the inhibition of mTORC1, sirolimus lowers MMP, oxygen consumption, and ATP levels. In addition, it reduces mitochondrial DNA copy number and many genes encoding proteins involved in oxidative metabolism [137, 138]. The mTOR signaling pathway also prevents islet cells from initiating authophagy [139]. Therefore, the inhibition of mTORC1 by sirolimus causes downregulation of T-lymphocytes while promoting authophagy and increasing the probability of initiating apoptosis. This has been demonstrated in a mouse model, whereby treatment with sirolimus induced an increase in membrane-bound light chain 3 (LC3-II), an early marker of authophagy, as well as a decrease in cell viability. In this experiment, sirolimus-mediated activation of authophagy was shown to be rescued by treatment with 3-methyladenine (3-MA), an inhibitor of authophagy [140].

Since sirolimus monotherapy is not sufficient to suppress islet graft rejection, it is often used with other immunosuppressants. Tacrolimus is one of the most commonly used immunosuppressant agents often used in combination with sirolimus. Tacrolimus immunosuppression blocks antigen-stimulated expression of genes such as IL-2 in T-cells, which is required for T-cell proliferation. It is generally assumed that the mechanisms involved in tacrolimus toxicity are similar to those of cyclosporine (CsA) by inhibition of mitochondrial ATP production and disruption of mitochondrial ion channels. However, some researchers have suggested that in an I/R model, tacrolimus protects neural tissues from adverse conditions such as overaccumulation of calcium, oxidative stress, cytochrome c release, and BAD phosphorylation [141, 142].

In clinical practice, sirolimus has been shown to increase basal and stimulated insulin secretion when maintained within a plasma-drug concentration target with reduced *β*-cell apoptosis [143]. When cultured with human islets, sirolimus decreases TNF-*α*, IL-1*β*, MCP-1, and macrophage inflammatory protein-1beta (MIP-1*β*) from impure islet preparations [144]. In the patients receiving sirolimus as preconditioning for islet transplant, an increase in fasting C-peptide levels and a decreased insulin requirement were observed [145]. In an *in vivo* rodent model, islet graft function was preserved in association with a pretransplant reduction in chemokines CCL2 and a dampened chemokine response posttransplant [146]. However, conflicting results have also been reported. Sirolimus has been found to impair metabolism secretion coupling by suppressing carbohydrate metabolism resulting in lower ATP levels, a slower glucose oxidation rate, and inhibited alpha-ketoglutarate dehydrogenase (AKGDH) [147]. In addition, sirolimus has been associated with a reduction in the amount of vascular endothelial grow factor (VEGF) that is released by the islets; a reduction in islet viability by blocking the VEGF-mediated survival pathways [148], as well as reduction in glucose-induced insulin secretion *in vivo* [149].

The detrimental effects of tacrolimus on *β*-cell function have been well documented including a reduction of insulin granules; decrease in insulin release; inhibition of insulin transcription as determined by RT-qPCR of insulin mRNA levels; reduction of glucokinase (I-GLK, hexokinase-4) activity using cell lines, rodent islets, and human islets [150–154]. One study has shown that tacrolimus reduces mitochondrial density and oxygen consumption at a pharmacologically relevant concentration without changes in either the rate of ATP production nor apoptosis. Microarray data indicate that tacrolimus modifies the pathways involving ATP metabolism, membrane trafficking, and cytoskeleton remodeling, indicating that tacrolimus causes mitochondrial dysfunction at the level of gene transcription and translation that causes a reduction in mitochondrial contents and respiration [155]. In 2009, one study compared the effect on sirolimus, tacrolimus, and mycophenolate mofetil (MMF) on glucose-induced insulin secretion in human islets and found out that all three of the drugs tested increased caspase-3 cleavage and caspase-3 activity. Tacrolimus has been shown to have acute and direct effects on insulin exocytosis, whereas MMF does not but still has impaired insulin secretion suggesting indirect effects on insulin exocytosis. Interestingly, exenatide (exendin-4), a GLP-1 agonist, can ameliorate impairments in insulin secretion caused by sirolimus, tacrolimus, and MMF [156].

In an *in vivo* rat model, sirolimus has been used with cyclosporine, showing that the combined treatment increases blood glucose and HbA1_**c**_levels, as well as the HOMA-R [fasting insulin (mU/mL) × fasting glucose (mmol/L)/22.5] index. When CsA was withdrawn, *in vivo* islet graft function was compromised when the patient was maintained with sirolimus [157]. One clinical case study reported that a patient who presented with sirolimus toxicity intolerance was converted to MMF with low-dose tacrolimus and maintained with a monthly infusion of daclizumab, showed good long-term islet graft function and improved renal function [158].

While a body of evidence seems to support the notion that most immunosuppressants currently used for islet transplant have at least some level of toxicity on islets and affect islet graft function, some conflicting results have been reported. Many contribute these incongruent observations to the selection of experimental models: *in vivo* versus *in vitro, *short-term versus long-term, cell line versus primary cells, animal versus human, and dosage difference.

In addition to immunosuppressant toxicity, the microenvironment of the islet engraftment site within the hepatic portal vein system has also been suggested as one of the main contributing factors associated with the loss of islet mass and function posttransplant. Within moments of islet implantation, a cascade of nonspecific inflammatory events known as instant blood mediated inflammatory reaction (IBMIR) are initiated which include activation of blood-mediated coagulation pathways [159] and release of proinflammatory cytokines (PIC) primarily from macrophage, Kupffer cells, and endothelial cells. It has also been suggested that cytokines produced by the immune system posttransplant can infiltrate pancreatic islets and serve as mediators of *β*-cell destruction [136]; as well as higher levels of immusuppressants that are directly toxic or damaging to the transplanted islets. The physical and chemical microenvironments of the hepatic portal system are also considered suboptimal for islet engraftment and survival: low oxygen tension and a high concentration of waste and nutrients may cause islet glucotoxicity and lipotoxicity. When islets are exposed to chronic hyperglycemic and hyperlipidemic environments, their overall function can be compromised including loss of glucose sensitivity, *β*-cell exhaustion, and glucotoxicity/lipotoxicity [160, 161]. Glucotoxicity and lipotoxicity are closely interrelated and complementary for their deleterious effects on *β*-cell function. Glucotoxicity involves several transcription factors and is in part mediated by chronic mitochondrial reactive species generation and accumulation. Lipotoxicity is probably mediated by the accumulation of a cytosolic signal derived from the fatty acid esterification pathway. Very few studies have been published that investigate islet grafts in the hepatic portal system due to the lack of good *in vitro* and *in vivo* models that can closely mimic this islet graft microenvironment. New alternative sites are currently being investigated including intramuscular, bone marrow, and the peritoneal cavity that could potentially decrease the stress to islets. While this topic is important for improving islet transplantation outcomes it has been previously reviewed in several other papers in greater depth [162, 163].

## 6. Summary

Recently, improvements in islet isolation techniques and the selection of optimal immunosuppression regiments have made human islet transplantation a viable clinical avenue in the treatment for T1DM patients. Inconsistent success rates are observed between short-term and long-term graft survival among centers and this could be related to the use of islet products of less than optimal quality and quantity. Mitochondrial functions are potentially compromised in each step of islet isolation and transplant process and subsequently can negatively influence islet engraftment and survival. The evidence presented in this review supports the following hypotheses: (i) mitochondrial dysfunction is a mediator for the onset and progression of islet I/R injury; (ii) potential therapeutics targeting mitochondrial processes, including energy metabolism, cytokine-induced inflammatory reaction, and free radical generation, will be promising in preserving islet function and viability. However, to confirm the role of mitochondrial cytoprotection in the isolated islets for transplant, it will be necessary to demonstrate these results in appropriate animal models and in a large cohort study of islet isolation by multiple centers to correlate clinical transplant outcomes. In addition, it is important to correlate a more sensitive and reliable islet viability and potency assay based on the evaluation of mitochondrial integrity in conjunction with other parameters to prevent the transplantation of a low quality islet preparations. Future efforts are also needed to better understand the underlying mechanisms of immunosuppressant-mediated islet toxicity and to discover a new generation of immunosuppressants that are less damaging to islets. Lastly, it is important to improve the islet transplantation process by decreasing islet loss potentially by finding a more suitable transplantation site in order to achieve consistent transplantation outcomes.

## Figures and Tables

**Table 1:** Oxygen carriers used in pancreas organ preservation and islet isolation.

Oxygen carriers	Benefits	Limitation	References
PFC	(i) Prevention of ischemia injury (ii) Improvement of islet yield and quality (iii) Inhibition of apoptosis by mitochondrial protection via oxygen loading (iv) Preservation of islet cell energetic status (v) Low islet oxidative stress (vi) Better *in vivo* islet graft function	(i) Inconsistent results despite several large-scale human trials (ii) May only be useful for marginal donors with prolonged ischemia (iii) Limited oxygen penetration into pancreas (iv) Poor water solubility (v) Indeterminate biosafety	[22–42]

PFD	(i) Preservation of islet ATP levels	(i) Only tested in animal model (ii) Impairment of insulin secretion	[43]

poly SFH-P	(i) Improvement of islet yield and quality (ii) Preservation of mitochondrial integrity (iii) No increase in oxidative stress (iv) Better *in vivo* islet graft function (v) High oxygen tension with oxygen saturation curve similar to RBCs	(i) Only in rodent model (ii) Warm-ischemia model used may not be applicable in a cold-ischemia model (iii) Product discontinued	[44]

**Table 2:** Major anti-oxidative and anti-inflammatory chemicals used in pancreas preservation, islet isolation, and islet culture.

Chemicals	Benefits	Limitation	References
AEOL10150 AEOL10113	(i) Antioxidative (SOS mimics) used in both isolation stage and culture (ii) Reduction of NF-*κ*B binding of DNA (iii) ↓IL-6,8; ↓MCP-1 (iv) Inhibition of the release of cytokines and chemokines and PARP activation (v) Protection of islets from oxidative stress	(i) No *in vivo* islet graft function available (ii) Has not been demonstrated by other groups	[58, 59]

Glutamine	(i) Benefits in both animal and human models during islet isolation (ii) Increasing of GSH levels (iii) Reduction of malondialdehyde and apoptotic cells (iv) Improvement of *in vivo *islet graft function	(i) Needs to be demonstrated by large-scale or multicenter studies (ii) Short-term effect due to instability and short half-life of glutamine (iii) Relationship with inflammatory cascade needs to be tested	[60–66]

SS-31	(i) Water-soluble antioxidative peptide with high islet penetration (ii) Specific mitochondrial targeting (iii) Preservation of mitochondrial polarization and reduced apoptosis (iv) Improvement of islet *in vivo* function	(i) Needs to be demonstrated by large-scale or multicenter studies (ii) Only tested in animal model	[67, 68]

NMMA Aminoguanidine N-acetyl cysteine Glutathione peroxidase	(i) Blockage of NO production via inhibition of iNOS in rodent and human islets (ii) Enhancement of islet antioxidant capability	(i) Only demonstrated *in vitro * (ii) The benefits for islet transplant need to be shown	[69–75]

Vitamins (D3, E, Riboflavin, C)	(i) Increased insulin secretion (ii) Higher islet viability (iii) Increased insulin gene expression (iv) Decreased lipid peroxidation	(i) Application in human islet isolation is limited (ii) No documented benefits on human islet receipts	[76]

Anakinra	(i) IL-1R antagonist via competitive inhibition (ii) ↓TNF-*α*; ↓IL-1*β*; ↓IFN-*γ* (iii) FDA approved as anti-inflammatory agent	(i) Not demonstrated in human islet isolation and transplant	[77–81]

Pan-caspase (ZVAD-FMK) and selective caspase inhibitor (zVD-FMK)	(i) Reduced islet loss during culture (ii) Improve islet graft function (iii) Reduced islet cell apoptosis	(i) No demonstrated benefits in human islet receipts	[82–84]

Prolactin	(i) Increased *β*-cell proliferation (ii) Increased insulin secretion (iii) Cytoprotection (iv) Improved islet engraftment (v) Increased islet revascularization (vi) Increased BCL2/BAX ratio (vii) Inhibition of caspase 8, 9 and 3	(i) Less understanding of mechanism (ii) Needs to be demonstrated by large-scale or multicenter studies	[85–88]

JNK inhibitor	(i) Increased islet yield (ii) Improved islet viability (iii) Improved *in vivo* graft function	(i) Limited information on human patients (ii) Large-scale study need to confirm	[89–93]

Pefabloc	(i) Efficient inhibition of serine protease activity (ii) Has been applied in all phases of isolation (iii) No interference with collagenase activity	(i) Controversial results on islet yield (ii) Inhibited insulin secretion *in vitro *	[94–98]

**Table 3:** Advantages and disadvantages of mitochondria-based islet potency and viability assays.

Assays	Advantages	Disadvantages	References
Newport Green + TMRE	(i) Low toxicity of Newport Green Dye (ii) *β*-cell specific (iii) Correlates with *in vivo *islet function	(i) Islet dissociation needed (ii) Nonstimulated static assay (iii) Complex instrumentation and setup required (iv) Difficult to quantify MMP (v) Not real time	[99, 100]

FluoZin-3 + TMRE	(i) *β*-cell specific (ii) FluoZin-3 has higher affinity (K_D_= 15 nM) for Zinc and higher quantum yield	(i) No correlation has been demonstrated with *in * *vitro* and *in vivo* islet function (ii) Nonstimulated static assay (iii) Complex equipment requirement and setup (iv) Difficult to quantify MMP (v) Not real time	[101, 102]

JC-1 + ROS	(i) Multiparametric assay (ii) Strong correlation of MMP with ROS (iii) Dynamic ROS assay (iv) Correlate with *in vivo* function	(i) Non *β*-cell specific (ii) Non-stimulated static assay of MMP (iii) Procedure complexity (iv) Intact islet assay for ROS but need islet dissociation for MMP (v) Not real time	[88, 103]

Multiparametric microfluidic assay (Rh123 + Fura-2AM + insulin kinetics)	(i) Multiparametric assay of key stimulus-secretion coupling factors (MMP: Rh123, Ca^2+^: Fura-2AM; Insulin: ELISA) (ii) Dynamic response to stimulator (iii) Intact islets (iv) Real time (v) High throughput	(i) Large-scale evaluation needed (ii) Moderate spatiotemporal resolution of the measured parameters	[104, 105]
